# Enrichment approach for unbiased sequencing of respiratory syncytial virus directly from clinical samples

**DOI:** 10.12688/wellcomeopenres.16756.1

**Published:** 2021-05-07

**Authors:** Jacqueline Wahura Waweru, Zaydah de Laurent, Everlyn Kamau, Khadija Said Mohammed, Elijah Gicheru, Martin Mutunga, Caleb Kibet, Johnson Kinyua, D. James Nokes, Charles Sande, George Githinji

**Affiliations:** 1 Epidemiology and Demographics, KEMRI Wellcome Trust Research Programme, Kilifi, KENYA, 237-80108, Kenya; 2 Biochemistry, Jomo Kenyatta University of Agriculture and Technology, Nairobi, Kenya, 62000-00200, Kenya; 3 Biochemistry and Biotechnology, Pwani University, Kilifi, Kenya, 195-80108, Kenya

**Keywords:** metagenomics, sequencing, SISPA, RSV, centrifugal processing, Endoh primers, DNase, RNA

## Abstract

**Background:** Nasopharyngeal samples contain higher quantities of bacterial and host nucleic acids relative to viruses; presenting challenges during virus metagenomics sequencing, which underpins agnostic sequencing protocols. We aimed to develop a viral enrichment protocol for unbiased whole-genome sequencing of respiratory syncytial virus (RSV) from nasopharyngeal samples using the Oxford Nanopore Technology (ONT) MinION platform.

**Methods:** We assessed two protocols using RSV positive samples. Protocol 1 involved physical pre-treatment of samples by centrifugal processing before RNA extraction, while Protocol 2 entailed direct RNA extraction without prior enrichment. Concentrates from Protocol 1 and RNA extracts from Protocol 2 were each divided into two fractions; one was DNase treated while the other was not. RNA was then extracted from both concentrate fractions per sample and RNA from both protocols converted to cDNA, which was then amplified using the tagged Endoh primers through Sequence-Independent Single-Primer Amplification (SISPA) approach, a library prepared, and sequencing done. Statistical significance during analysis was tested using the Wilcoxon signed-rank test.

**Results:** DNase-treated fractions from both protocols recorded significantly reduced host and bacterial contamination unlike the untreated fractions (in each protocol p<0.01). Additionally, DNase treatment after RNA extraction (Protocol 2) enhanced host and bacterial read reduction compared to when done before (Protocol 1). However, neither protocol yielded whole RSV genomes. Sequenced reads mapped to parts of the nucleoprotein (N gene) and polymerase complex (L gene) from Protocol 1 and 2, respectively.

**Conclusions:** DNase treatment was most effective in reducing host and bacterial contamination, but its effectiveness improved if done after RNA extraction than before. We attribute the incomplete genome segments to amplification biases resulting from the use of short length random sequence (6 bases) in tagged Endoh primers. Increasing the length of the random nucleotides from six hexamers to nine or 12 in future studies may reduce the coverage biases.

## Introduction

Unbiased sequencing of bacterial, fungal and viral communities has been used to characterize the microbial diversity in nasopharyngeal samples and aid in explaining diseases of unknown aetiologies ( Camelo-Castillo *et al.*, 2019; Geliebter *et al.*, 2020; Lu *et al.*, 2020). Unlike targeted sequencing, unbiased sequencing strategies do not require prior knowledge of pathogens present in a sample thus eliminating relative abundance biases inherent to targeted sequencing ( Camelo-Castillo *et al.*, 2019; Graf *et al.*, 2016). While bacterial and fungal metagenomics studies make use of the 16S and ITS (internal transcriber spacer) conserved markers for bacterial and fungal community amplification, respectively, viral communities lack conserved markers within viral families ( Camelo-Castillo *et al.*, 2019; Conceição-Neto *et al.*, 2015; Geliebter *et al*., 2020), making random priming also termed as Sequence Independent Single Primer Amplification (SISPA), a promising metagenomics strategy ( Djikeng *et al*., 2008).

SISPA was first developed by Reyes & Kim (1991), and entails the use of oligonucleotides consisting of random nucleotides on the 3’ end and a 5’ defined tag sequence that is mainly used for subsequent amplification ( Chrzastek *et al.*, 2017). Though SISPA has previously proved effective in metagenomics studies, it results in preferential sequencing of the most abundant nucleic acid material in a nasopharyngeal sample; mainly host and bacteria ( Djikeng *et al.*, 2008; Goya *et al*., 2018). To counter this, methods often incorporate physical and enzymatic virus enrichment steps including centrifugal filtration and DNase treatment ( Conceição-Neto *et al*., 2015; Goya *et al.*, 2018; Thurber *et al*., 2009). SISPA, centrifugal filtration and DNase treatment were employed in several studies ( Chrzastek *et al.*, 2017; Goya *et al.*, 2018; Lewandowski *et al.*, 2020) and deemed effective in enhancing viral read representation and in reducing bacterial and host contamination.

We endeavored to develop a metagenomics protocol for respiratory syncytial virus (RSV); a leading cause of lower respiratory tract infections among children under the age of five. RSV accounts for approximately 33.1 million cases and an estimated 3.2 million hospitalizations globally per year among children under the age of five years ( Shi *et al.*, 2017). Roughly 48,000-74,500 in-hospital child deaths annually are attributed to RSV infections ( Shi *et al.*, 2017). The virus also causes high morbidity and mortality among immunocompromised individuals and the elderly ( Englund *et al.*, 1991; Lee *et al.*, 2013). The genome of the virus is a 15.2 kb non-segmented, negative-sense, single-stranded ribonucleic acid (RNA) virus ( Mufson *et al.*, 1985) belonging to the order *mononegavirales*, *pneumoviridae* family and the *Orthopneumovirus* genus ( Rima *et al.*, 2017). Here, we utilized centrifugal filtration ( Thurber *et al*., 2009), DNase-treatment ( Peret *et al*., 1998) and SISPA ( Nguyen *et al.*, 2016), as virus enrichment methods for RSV sequencing using the Oxford Nanopore Technology (ONT) MinION device: an affordable, long read and portable real-time single molecule sequencing device with potential for virus metagenomics studies ( Lewandowski *et al*., 2020; Miani *et al*., 2020).

## Methods

### Study samples

Thirty-two nasopharyngeal swabs (NPS) collected between January 2012 and December 2015 from children under the age of five years presenting to the Kilifi County Hospital with clinical symptoms of severe pneumonia were selected for this study using the purposive sampling approach. All NPS samples used in this study were collected upon hospital admission by the clinicians on duty, stored in a universal transport media, kept at 8°C in an ice packed cool box, and transported to KEMRI-Wellcome Trust Research Programme laboratories four hours after collection where they were stored at -80°C. For samples to be included in this study, they had to have been confirmed positive for RSV using the indirect immunoflourescent antibody test (IFAT) and reverse transcription polymerase chain reaction (RT-PCR) method and recorded high viral load as identified by low cycle threshold scores (Ct < 24). In addition, samples included here had to have been sequenced using MiSeq (Illumina) by targeted amplification and full genomes obtained ( Agoti *et al*., 2015; Otieno *et al*., 2018). We excluded samples with low cycle thresholds (Ct > 24) whose full genomes had not been unravelled before.

### Ethical considerations

The study was ethically approved by the Kenya Medical Research Institute (KEMRI) Scientific and Ethics Review Unit (SERU #3103). Written informed consent had been collected from all the patient caregivers before using the samples for this study.

### Sample processing

**Figure 1.**
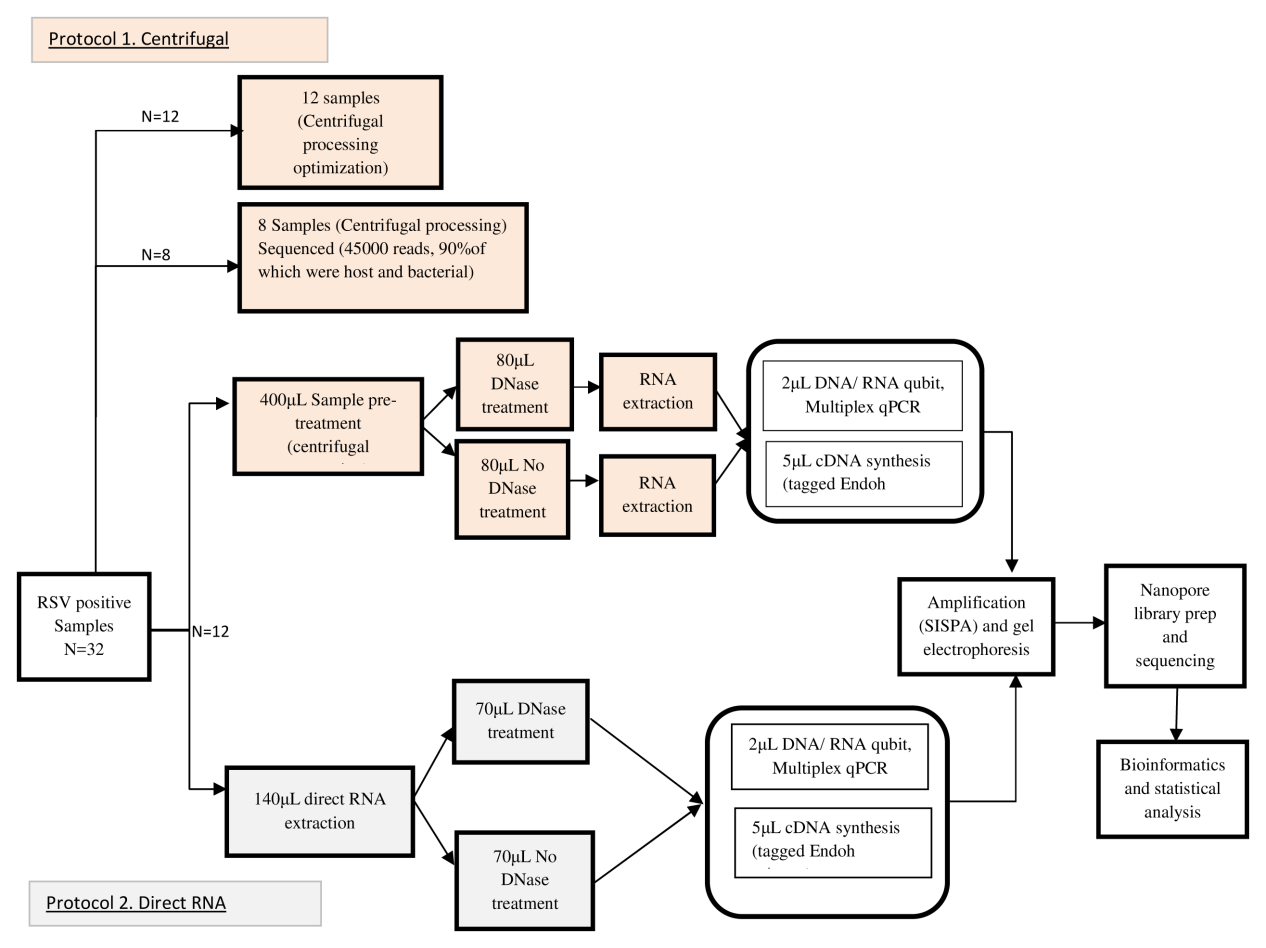
A flow chart representing the experimental setups tested in this study. In total, 12 samples were selected and divided into two fractions: the first underwent centrifugal processing (Protocol 1) and the entire workflow is represented by the upper part of the flow chart while the second underwent direct RNA extraction (Protocol 2) and the entire workflow of the fractions treated using the approach is represented on the lower part of the flow chart. The arrows indicate the process from one step to the next.

Each of the processes for the two protocols is set out in the flow diagram depicted in Figure 1.

### Protocol 1: Centrifugal processing approach

***Optimization.*** A set of 12 RSV positive samples were used at first to optimize the centrifugal pre-processing protocol. The protocol involved centrifugation of 400μl of sample at 8000 rpm for 5 minutes, which resulted in a pellet constituted mainly of the dense host and bacterial content. A volume of 350μL supernatant was collected and transferred to the 3kD Scientific Centrifugal Filter (Thermo Fischer), for centrifugal filtration for one hour at 14,000rpm to recover, separately, concentrates and filtrates. RNA was extracted from each of the three sample fractions (concentrate, filtrate and pellet from centrifugal processing) obtained from the 12 samples using the QIAmp viral RNA kit (QIAGEN) according to the manufacturer’s instructions. Briefly, samples were lysed under high denaturing conditions to inactivate RNases and to enhance the isolation of intact viral RNA, buffering conditions adjusted to provide optimum binding of the RNA to QIAMP membrane, contaminants washed away and high quality RNA precipitated and eluted in RNase free buffer ready for subsequent steps. The effectiveness of the pre-processing steps was assessed by performing RNA HS (high sensitivity) qubit, multiplex RT-PCR and IFAT. Quantity and quality of the RNA extracts were determined using Qubit RNA HS assay. RT-PCR assays for RSV ( Hammitt *et al.*, 2011; Venter *et al.*, 2011) were used to quantify the viral load in the three sample fractions. The differences in the viral Ct scores between the concentrate and the pellet were used to infer the extent of host contamination. IFAT using RSV DFA kit Light Diagnostics™ was further used to inform the extent of host contamination between the pellet and the concentrate by observing the intensity of red and green fluorescence (red fluorescence represents host cells while green represents viruses) in the two fractions. Bacterial contamination in the concentrate was determined using conventional PCR, with primers that target the V3 and V4 region of the 16S ribosomal RNA (rRNA). Amplified PCR products were visualized in a 2% agarose gel.

***Sequencing***. All the sample volumes used during the centrifugal processing optimizations were depleted prompting us to select 8 additional RSV positive samples to assess the effectiveness of the approach during sequencing. We took the 8 additional samples through centrifugal processing approach, RNA extraction, cDNA synthesis, SISPA, library preparation and sequencing. However, only 45,000 reads were obtained from the sequencing run, 90% of which were host and bacterial, hindering further analysis. This prompted us to adopt a DNase treatment step after the centrifugal processing. Since the sample volumes for the eight samples also had depleted, we selected 12 additional samples. We used 400μL of each of the samples and took them through centrifugal processing and the resulting concentrate was divided into two equal fractions: the first was DNase treated to remove the genomic DNA concentration from our RNA using TURBO DNase (Thermo Fischer) while the second was not, followed by RNA extraction.

### Protocol 2: Direct RNA extraction approach

From the remaining volume of the 12 samples, we used 140μL from each with the direct RNA extraction protocol. This involved extracting RNA from the samples without a prior physical or enzymatic enrichment step using QIAmp viral RNA kit (QIAGEN) according to the manufacturer’s instructions. The resulting RNA was divided into two equal fractions, the first was DNase treated to also remove genomic DNA from our RNA of interest using TURBO DNase (Thermo Fischer), while the second was not.

### Sequence independent single primer amplification (SISPA)

First-strand cDNA was synthesized in a 20μl reaction from 5μl viral RNA extracts from both protocols using the Superscript III reverse transcriptase kit (Thermo Fischer Scientific), according to the manufacturer’s instructions and using the FR26-Endoh primers ( Nguyen *et al*., 2016). Briefly, the FR26-Endoh primers; created by replacing the 3’ end of the FR26RV-N with those of 96 non ribosomal hexanucleotides designed by Endoh ( Endoh *et al*., 2005), were added to the template along with nuclease free water and deoxynucleoside triphosphate (dNTPs), and the mix heated at 65°C for 5 minutes. After heating, the mix was chilled on ice for one minute and the first strand synthesis mix constituted of first strand buffer, DTT, superscript III and RNaseOUT added, followed by incubation at 55°C for 40 minutes and inactivation of the reaction at 70°C for 15 minutes. Klenow fragment 3’-5’ exo (NEB) was used to convert the first-strand to second-strand cDNA: 20μl of the first-strand cDNA mixture was incubated at 37°C for 90 minutes in the presence of dNTPs, nuclease-free water, and 10X buffer. The RSV RT-PCR assay was used to confirm cDNA formation by excluding the RT step during the PCR cycle because the reverse strand had been generated during the cDNA synthesis step.

The FR20RV primer and Q5 PCR kit (NEB) were then used to amplify 13μl of the double-stranded cDNA as follows: 98°C for 30s, 38 cycles of 98°C for 10s, 55°C for 30s and 72°C for 1 min. This PCR was run twice to complete any partial amplicons resulting from used up dNTPs and primers in the first amplification. PCR products were visualized in a 1% gel and purified using Agencourt AMPure XP beads (Beckman Coulter).

### Nanopore library preparation and sequencing

We prepared our library by multiplexing up to 24 end-repaired samples using the Oxford Nanopore 1D ligation sequencing kit (SQL-LSK 109). In brief, all the samples were barcoded using the native barcoding kits (EXP-NBD 104 and EXP-NBD 114), and the enzyme T4 ligase. After barcoding, the samples were washed using the AMPure XP beads (Beckman Coulter), and eluted using an elution buffer. 1ul of barcoded samples were used in quantification using the Invitrogen Qubit double stranded DNA HS kit (Thermo Fisher) and the obtained concentrations used during the normalization process. Normalization was done to ensure that equimolar amounts of the barcoded samples were picked when pooling the samples together. To the pooled barcoded samples, adapter ligation was done using Adapter mix II (AMII), Nebnext Ultra II ligation master mix and Nebnext ligation enhancer. After a 10min incubation to enhance the adapter ligation process, a clean-up using the AMPure XP beads and short fragment buffer (SFB) in place of ethanol was done. The adapter ligated samples were eluted using 15ul elution buffer, 2ul of which was used during quantification using qubit. A library mix containing 12ul of the DNA, 25.5ul of the loading beads and 37.5ul of the sequencing buffer was prepared and loaded on a QC-ed R9.4.1 flow cell (FLO-MIN106) and sequencing performed using MinKNOW software (version 19) for 12 hours.

### Bioinformatics analysis

The reads generated from both protocols were taken through bioinformatics analysis using open source tools other than for the Guppy base-calling software (version 3.1.5, ONT technologies). An alternative open source base calling tool is Scrappie ( https://github.com/nanoporetech/scrappie), a technology illustrator also developed by ONT community. The output FAST5 files were base called and de-multiplexed using Guppy version 3.1.5 and then quality checked (QC) using PycoQC (version 2.5.0.23) ( https://anaconda.org/bioconda/pycoqc/files?version=2.5.0.23) ( Leger & Leonardi, 2019) after which taxonomic classification using Kraken2 (version 2.0.9beta) ( https://anaconda.org/bioconda/kraken2/files?version=2.0.9beta) ( Wood *et al.*, 2019) was done. All the reads that passed QC (Phred score >7) test were then mapped to the corresponding 12 RSV references generated from Illumina using Minimap2 (version 2.17) ( https://anaconda.org/bioconda/minimap2/files?sort=ndownloads&sort_order=desc&version=2.17) ( Li, 2018) and the resulting SAM files converted to a BAM file, sorted and indexed using SAMtools (version 1.7) ( https://anaconda.org/bioconda/samtools/files?version=1.7) ( Li *et al*., 2009). Sorted bam files were visualized using Integrated Genomics Viewer (IGV) (version 11.0.1) ( https://software.broadinstitute.org/software/igv/) ( Thorvaldsdóttir *et al.*, 2013) to determine the regions they mapped to in the genome. We then searched the Endoh primers against a centroid genome generated from the consensus Illumina reads using Vsearch cluster (version 2.15.0) ( http://phoenix.yizimg.com/wulj2/vsearch) ( Shen *et al*., 2016), and the regions to which the primers mapped located using Seqkit locate (version 0.13.2) ( https://anaconda.org/bioconda/seqkit/files?version=0.13.2&type=) ( Rognes *et al.*, 2016). All statistical analyses to generate the bar graphs and boxplots presented in the results section were done in R version 3.6 ( R Core Team, 2019).

## Results

### Protocol 1: Centrifugal processing approach optimization

**Figure 2.**
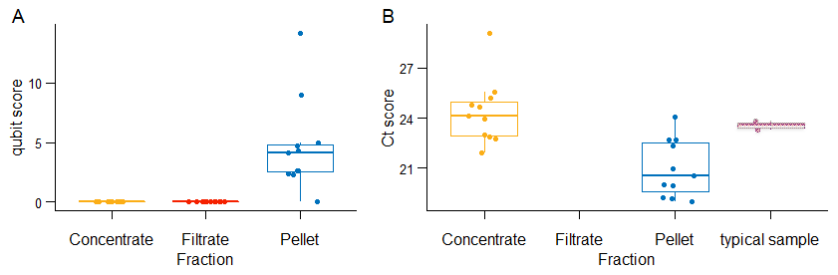
**A.** Boxplot of the qubit scores from eight centrifugal processed samples against sample fraction. **B.** A boxplot of RSV RT-PCR cycle threshold scores of twelve samples against the sample fractions (concentrate, filtrate and pellet). The colours represent the sample fractions. Filtrate in panel B is undefined, indicating a Ct value >=40.

**Figure 3.**
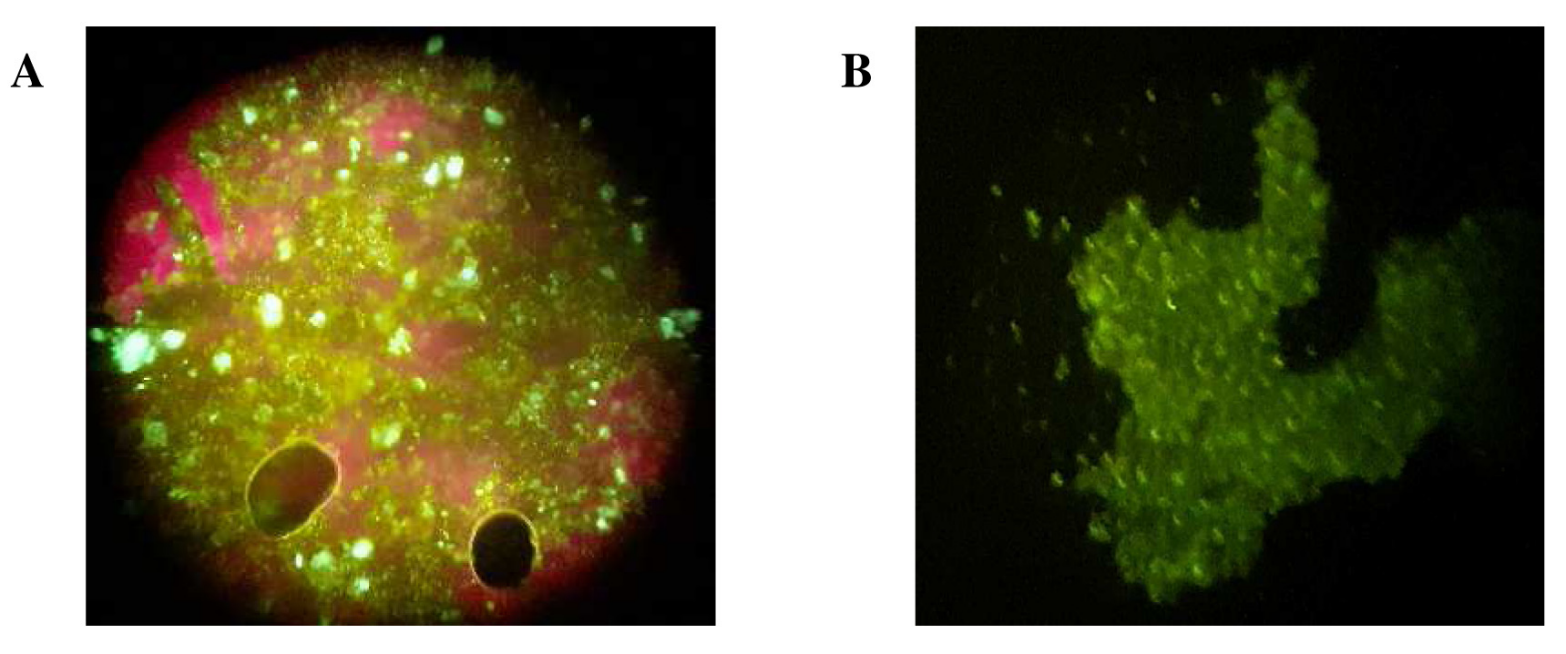
IFAT images of the pellet ( **A**) and the concentrate ( **B**). Red fluorescence in the pellet represents host cells while green fluorescence in both the pellet and the concentrate represents RSV particles.

***3.1.1: Optimization.*** After comparing the RNA Qubit scores, cycle threshold (Ct) scores and IFAT images from the concentrate, filtrate and pellet ( Waweru *et al*., 2021), we observed that nucleic acid content in the concentrate and filtrate was undetectable compared to the pellet ( Figure 2A). The filtrate was RSV negative suggesting little or no virus loss during centrifugal filtration while the pellet had a lower Ct score than the concentrate suggesting more viral content in the pellet relative to the concentrate ( Figure 2B). Samples taken through direct RNA extraction as described in Protocol 2 but not treated with DNAse termed as typical RSV positive samples here, had comparable Ct scores to the concentrates ( Figure 2B). The concentrate’s low RNA qubit scores and reduced viral load implied reduced host contaminants as compared to the pellet, as also confirmed by IFAT, where, IFAT images from the concentrate and the pellet indicated that in addition to the green fluorescence signifying virus particles, the pellet had more red fluorescence indicative of host cells as compared to the concentrate, as shown in the images in ( Figure 3). The differences in the red fluorescence is indicative of differences in the degree of host contamination in the two sample fractions (pellet > concentrate).

**Figure 4.**
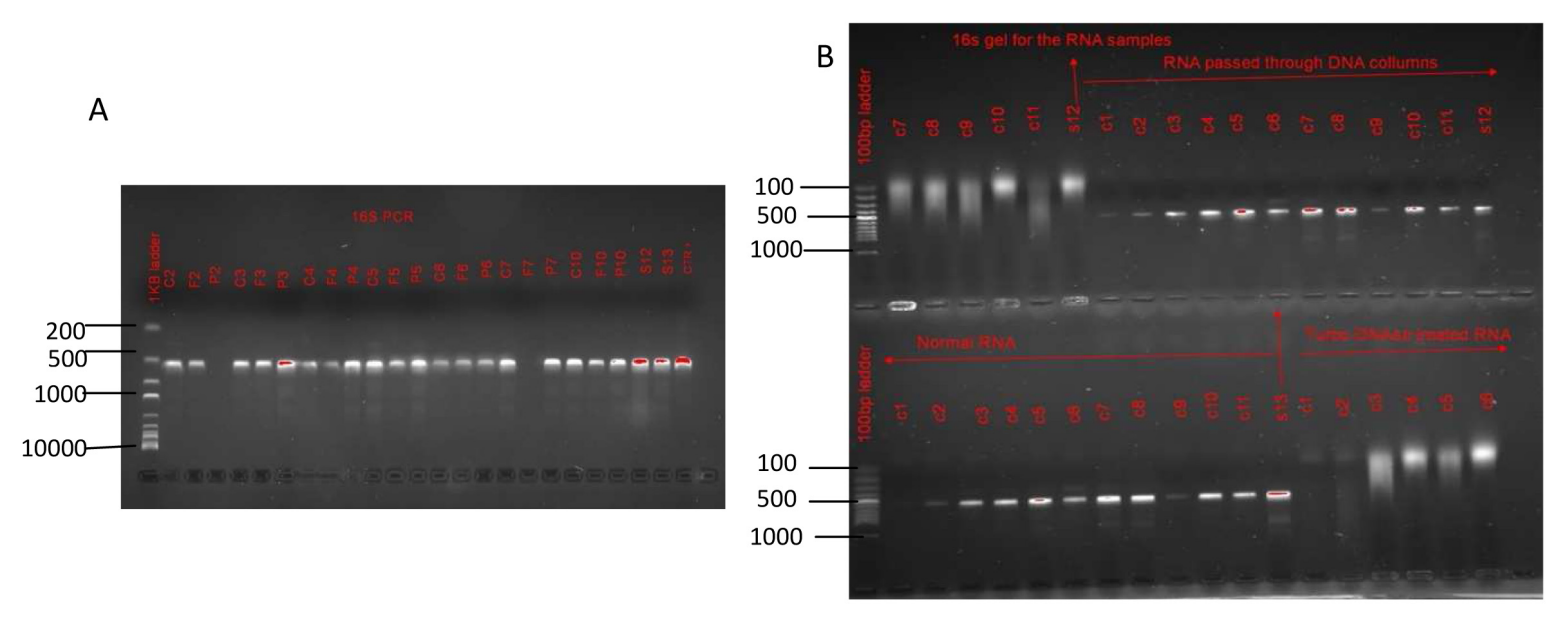
16s rRNA gel images. Gel image **A** demonstrates bacterial contamination in the various sample fractions. Gel image **B** is an illustration of the impact of DNase treatment and DNA columns in reducing bacterial contamination.

**Figure 5.**
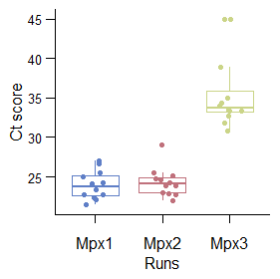
A boxplot of Ct values against runs which demonstrates the effect of DNase treatment in reducing viral load content in the concentrate. Mpx1 represents the Ct values when selecting the samples, Mpx2 the Ct scores from the concentrates after centrifugal processing and Mpx3 the Ct values after treating the concentrates with DNase.

An analysis of the 16S rRNA PCR results indicated that the concentrate, which was the main sample fraction of focus in this study, still contained a lot of bacterial contamination ( Figure 4A). Alternatives to reduce the contamination entailed adoption of DNase treatment using Turbo DNase or passing the extracted RNA through DNA columns. Of the two alternatives, DNase treatment appeared most effective in reducing the extent of bacterial contamination as compared to the use of DNA columns ( Figure 4B). However, treating the concentrates with DNase reduced the viral load initially present in the concentrates, as confirmed by a rise in Ct scores in the concentrates treated with DNase ( Figure 5). This observation prompted us to treat the concentrates with DNase just before RNA extraction, a strategy that was deemed effective at reducing host contaminants while protecting the viral genomes from digestion, and enhancing viral reads representation in the final metagenomics dataset in a study by ( Lewandowska *et al.*, 2017).

### Sequence independent single primer amplification (SISPA)

**Figure 6.**
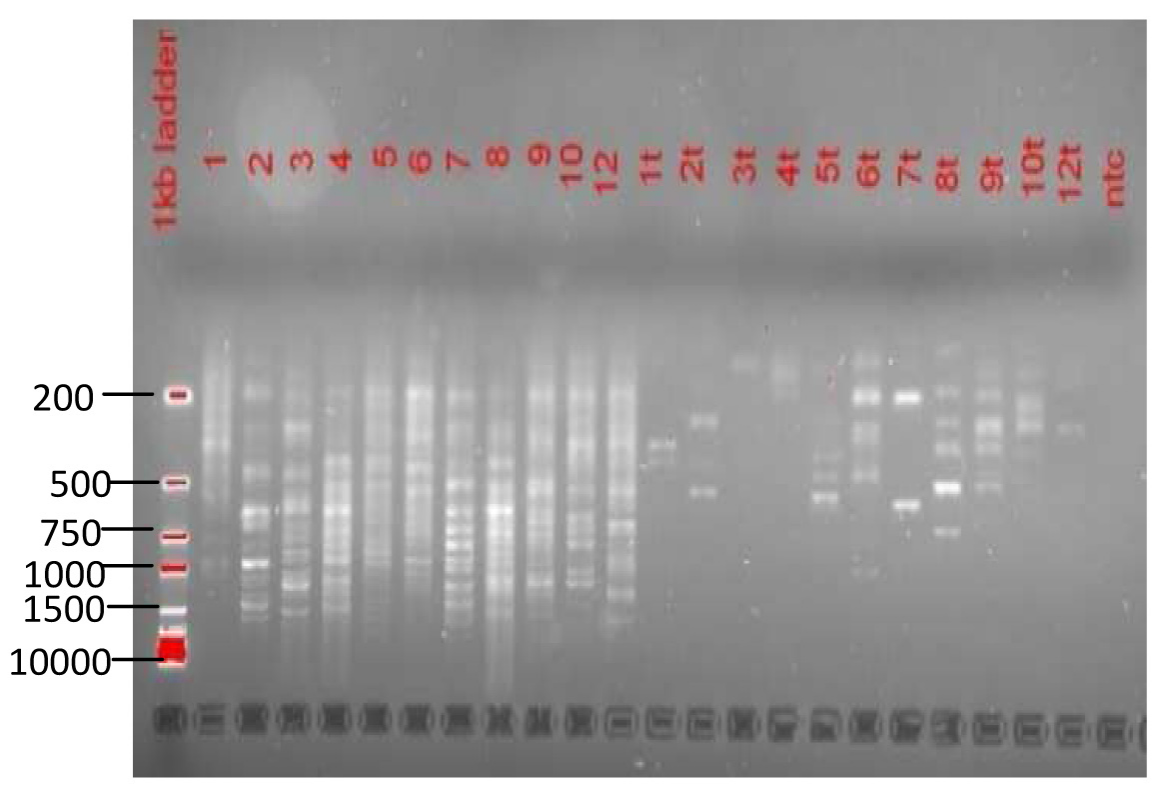
A gel image after performing SISPA. DNase treated sample fractions are denoted with a‘t’ after the sample ID while traces with the sample ID alone denotes the untreated fractions.

Random amplification using SISPA resulted in PCR products of varying lengths ranging between 250 bases to 1500 bases. The varying PCR products were more prominent in the samples not treated with DNase ( Figure 6). The varying lengths in the band sizes demonstrated that the SISPA approach was successful in untargeted amplification of nucleic material present in each sample.

### Protocol 1: Centrifugal processing results

**Figure 7.**
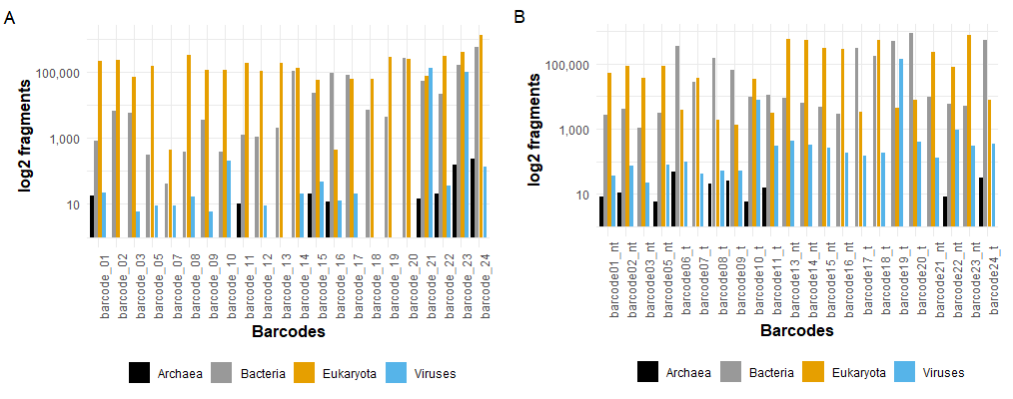
A graphical representation of the domains present in the obtained reads per barcode. Panel **A** represents the domains present in the sample fractions that underwent centrifugal processing (Protocol 1), while panel **B** represents the domains present in the sample fractions that underwent direct RNA extraction (Protocol 2).

**Figure 8.**
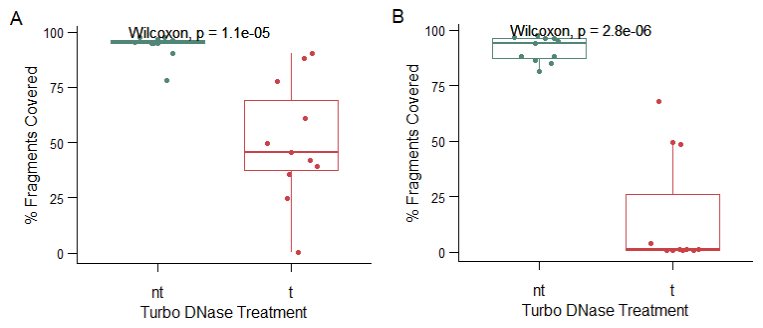
A boxplot of the distribution of host reads between the DNase treated (t) and the non-treated (nt) sample fractions in sample fractions that were processed using Protocol 1 in panel **A** and those that were processed with Protocol 2 in panel **B**.

**Figure 9.**
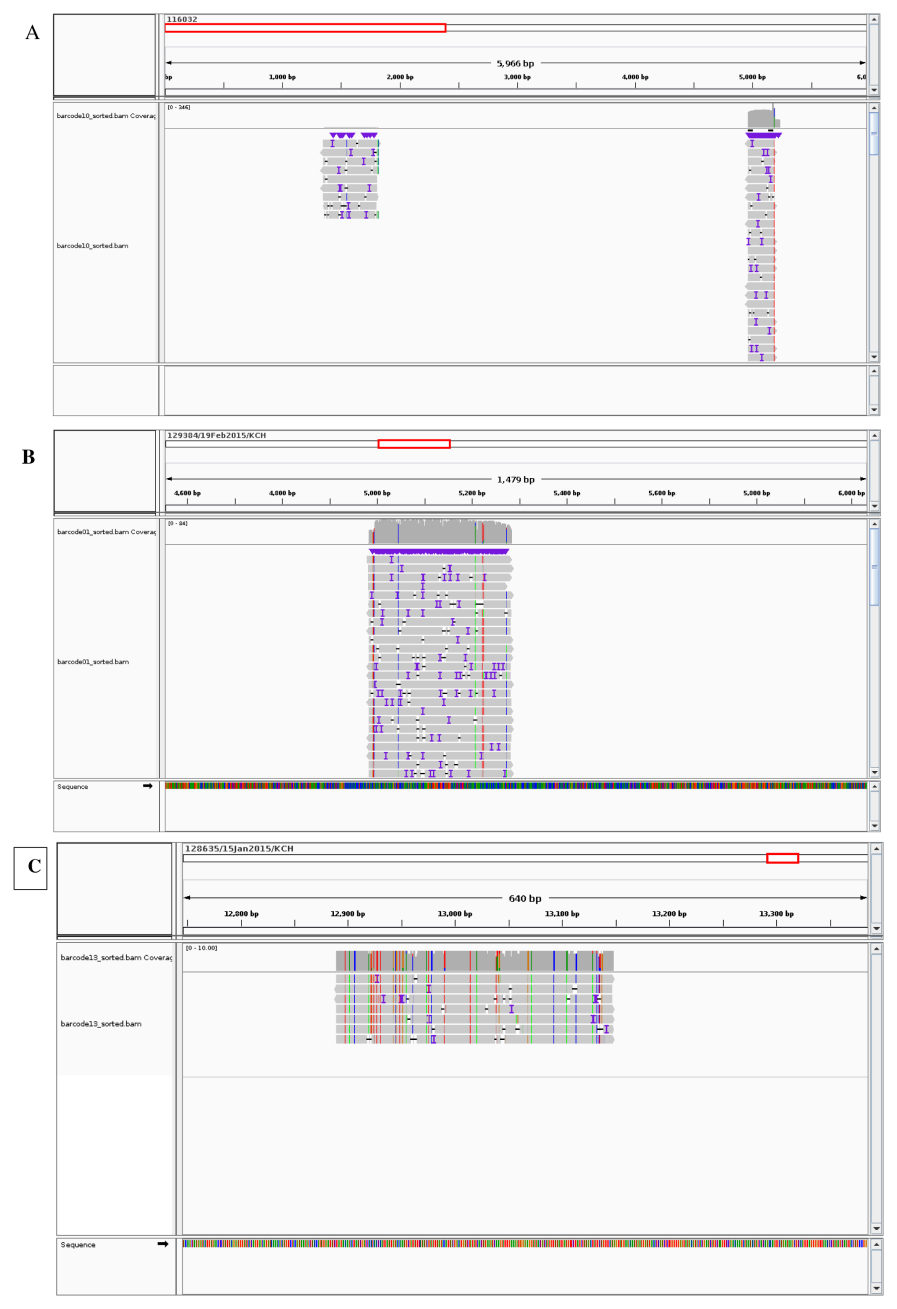
Screen shots of the regions to which RSV reads mapped using Illumina consensus references. ( **A**) illustrates the region to which the reads from Protocol 1 mapped: part of the N gene, with some additional reads mapping to part of the G gene while ( **B**) and ( **C**) illustrates the regions to which the reads from the Protocol 2 mapped: part of the RSV G and L genes respectively

We recovered 8.2 million reads from this protocol, 7.2 million of which passed quality check (QC) with their median read quality being 11.11. Taxonomic classification of all the reads that passed QC from this protocol using Kraken2 indicated that the most abundant domains were Eukaryota and Bacteria as compared to those from viruses ( Figure 7A). A comparison of the extent of host and bacterial contamination between the DNase treated and untreated sample fractions indicated that DNase treated sample fractions had significantly lower contamination extents as compared to the untreated ( *p*= 0.000011), ( Figure 8A). No full RSV genome was recovered from this protocol and the sequenced reads mainly mapped to part of the N gene ( Figure 9A), with the total number of sequenced bases being roughly 470, spanning from around 1350 bases to around 1800 bases. Additional reads in samples labelled with barcodes 10 and 21 from the same protocol mapped to part of G and L genes respectively with the total number of sequenced bases being 271 and 266 spanning the regions between 4970 to 5245 and 12900 to 13166 respectively.

### Protocol 2: Direct RNA extraction results

This protocol yielded 8.2 million reads, 6.8 million of which passed quality check (QC). The median read quality for all the reads that passed QC was 10.33. Taxonomic classification of the reads that passed QC using Kraken2 indicated that the most abundant domains from this protocol were also Eukaryota and Bacteria as compared to those from viruses ( Figure 7B). A comparison of bacterial and host contamination extents between the DNase treated and untreated sample fractions from this protocol also showed significantly lower contamination extents in the DNAse treated fractions as compared to the untreated ( *p*= 0.0000028) ( Figure 8B). Nonetheless, no full RSV genome was recovered from this protocol either with reads from barcodes 01 and 06 mapping to part of the G gene ( Figure 9B), with the total number of sequenced reads being roughly 305 spanning the regions between 4900 to roughly 5200. Reads from barcodes 13-24 on the other hand mainly mapped to part of the L gene ( Figure 9C) with the total number of sequenced bases being roughly 258 spanning from around 12890 bases to 13160 bases.

### Comparison of centrifugal processing and direct RNA extraction protocols

**Figure 10.**
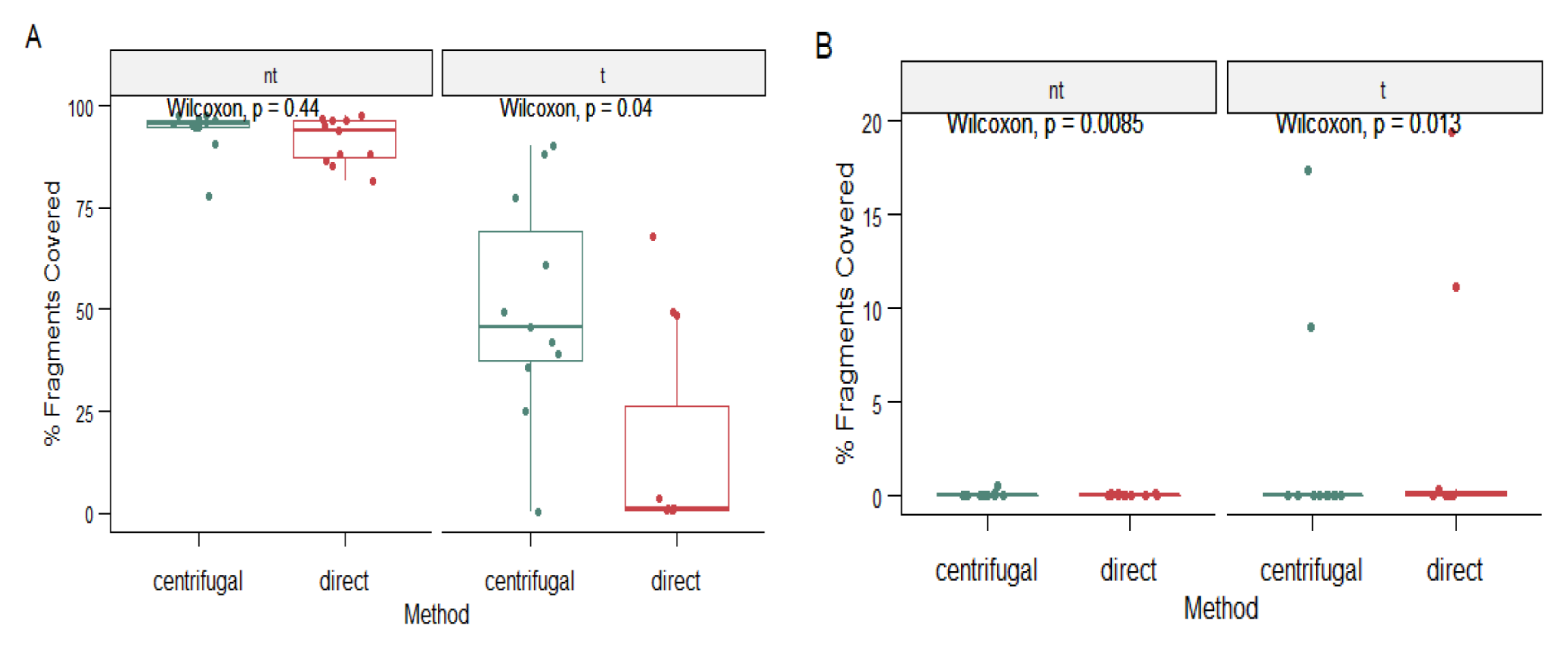
Box plot in panel **A** shows the comparison of proportion of host reads between the two protocols while that in **B** shows the proportion of RSV reads between the treated and untreated sample fractions with those treated with Protocol 1 labelled centrifugal and those processed with Protocol 2 labelled direct.

Given that the same 12 samples were sequenced in both protocols; we observed that the regions that the reads span varied per run with the average percentage genome coverage in reads that underwent centrifugal processing being 3% and 1% for those that underwent direct RNA extraction. In addition, when we compared the proportions of host reads between the DNase treated and untreated fractions from the two protocols, we observed that there was a significant difference in the treated fractions ( *p* = 0.04), with greater reductions in those extracted using Protocol 2, while there was no significant difference in the untreated fractions ( *p* = 0.44) between the two protocols Figure 10A. When we compared RSV reads yield from the two protocols, we observed a significant difference in the proportion of RSV reads between the DNase treated ( *p* = 0.013) and untreated fractions ( *p* = 0.0085) from both experimental setups with the more RSV reads in the DNase treated and directly extracted samples compared to those that underwent centrifugal processing ( Figure 10B).

## Discussion

In this study, centrifugal processing, nuclease treatment using DNase and random amplification using SISPA were tested for metagenomics sequencing of clinical respiratory viruses in RSV positive specimens. The results from the sample extraction optimization step demonstrated that most of the viruses were embedded in the pellet, which was highly abundant in host cells ( Figure 3A). Centrifugal processing recovered freely floating viruses in the concentrate consisting of reduced host cells, although its viral load was reduced. However, centrifugal processing showed little impact in reducing bacterial contamination as confirmed by 16s rRNA PCR ( Figure 4A), but DNase treatment was deemed most effective at reducing the extent of bacterial contamination but at the expense of reduced viral content ( Figure 5). Despite these processes, we were unable to recover full RSV genome from either protocol.

A comparison of our findings in Figure 2 and Figure 3 showed congruence with what has been done previously since Hall *et al.* (2014), Goya *et al*. (2018) and Thurber *et al.* (2009) showed that the adoption of centrifugal filtration prior to RNA extraction at moderate speeds helped in reducing host contaminants and increased the recovery of viruses. Thurber *et al.* (2009) demonstrated that centrifugal processing was a suitable sample pre-treatment process because viruses are encapsulated enabling them to withstand concentration without resulting in the degradation of the nucleic material. Nevertheless, Hall *et al.*, (2014) cautioned on the speed and time set while running centrifugal processing since the process results in reduced viral load and the loss was more significant with increased centrifugation speeds and time due to the continuous precipitation of the particles including viruses present in a sample. Low centrifugation speeds, on the other hand, had no impact in reducing host contaminants ( Hall *et al.*, 2014).

This study further demonstrated that the use of centrifugal processing did not reduce the amount of bacterial contamination in the samples ( Figure 4). Hall *et al*., (2014) indicated that though the centrifugal filters reduced bacterial contamination in a clinical sample, their efficiency in facilitating bacterial loads reduction in a specimen was reduced. DNase treatment as recommended by metagenomics studies by Goya *et al.* (2018), Allander *et al.* (2001) and Rosseel *et al.* (2015) was deemed most effective at improving the identification of viruses and reducing the extent of bacterial and host contaminants. The highly abundant host and bacterial reads compared to viruses in our dataset even after DNase treatment confirmed how challenging it is to deplete the two major contaminants.

Reference mapping analysis from this study indicated that no complete RSV genome was recovered from either of the two protocols, with the identified genomic segments spanning varying regions of the genome from both protocols. These observations suggest an incidence of preferential amplification of the most abundant regions of the genome when SISPA was done. Rosseel *et al*., (2013) and Victoria *et al*., (2009) made closely similar observations and reported that the SISPA technique introduced coverage depth distribution bias. In their studies, Rosseel *et al.* (2013) and Victoria *et al.* (2009) observed gaps in areas of low complexity and exaggerated sequence depths in the preferentially amplified regions. Rosseel *et al.* (2013) attributed the SISPA coverage depth bias to annealing biases introduced by the primer used, where the annealing of the random hexamers is enhanced when some nucleotides termed as annealing sites specific to the 5’ amplification tag (designed for PCR amplification) assist the random hexamers at the 3’ end in annealing during first strand synthesis. In our study, we also speculate that the uneven distribution of the reads across the RSV genome and the variation in the regions that the reads span per run was as a result of part of the tag annealing to the genomic sequence. Of interest in this study was the random primers named 59, 87 and 92 which had some bases on the tag annealing to the centroid sequence and resulting to the over-amplification of the main regions that our reads span ( Table 1). The primer labelled 87 specifically which presumably amplified part of the N gene recovered in this study, had six bases constituting the tag annealing to our centroid genome.

**Table 1.** A tabulation of the primers that could have played a role in preferential amplification of some genomic regions of the RSV region in our study.

sequence	Primer name	Pattern	Strand	Start	End	Matched
113388	Primer 59	CATATTG	-	12879	12885	CATATTG
113388	Primer 87	GATATCATGTTA	+	1355	1366	GATATCATGTTA
113388	Primer 92	CCATACT	+	4974	4980	CCATACT

Additionally, the results from this study demonstrated that significant depletion of host and bacteria reads from viral reads was dependent on whether DNase was done prior to RNA extraction or after RNA extraction. Significant reduction in contamination levels was more evident in samples that were extracted using the direct RNA protocol and treated with DNase after RNA extraction as compared to those that underwent centrifugal processing and their concentrate treated with DNase prior to RNA extraction. A high number of host reads after centrifugal processing and DNase treatment, as seen in this study, could be attributed to ribosomes held within the concentrate ( Rosseel *et al.*, 2015). Rosseel *et al.* (2015) indicated that pre-treating the concentrate with DNase prior to RNA extraction had no impact on ribosomal RNA as they stayed protected from the nucleases and were released during the RNA extraction process, resulting in high host reads relative abundances after extraction.

In summary, this study demonstrates that although physical virus enrichment approaches such as centrifugal processing help in enriching for the viruses in a viral metagenomics dataset, they cannot be used independently in metagenomics studies. Large amounts of host and bacterial reads are still recovered even after physical enrichment thus making it paramount to include an enzymatic depletion step using DNase, although at the expense of decreasing the virus component. DNase activity should be done after RNA extraction to achieve the best DNase activity in depleting host and bacterial contaminants. During random priming, it is important to consider the length of the random primers being used to avoid preferential amplification biases introduced by using short hexamers in this study. Increasing the length of the random nucleotides from six hexamers to 9 or 12 in future studies is merited as FR20RV-9mer or FR20RV-12mer have been shown to be more stable and enhanced the chance of their equal distribution across the genome ( Rosseel *et al*., 2013).

## Data availability

Harvard Dataverse: Replication Data for: Enrichment approach for unbiased sequencing of respiratory syncytial virus directly from clinical samples. https://doi.org/10.7910/DVN/28LOAI ( Waweru *et al*., 2021).

This project contains the following underlying data:

-    Data.zip (Raw datasets)
-    Boxplots_script.R; taxonomic_analysis.R (The analysis scripts)
-    Bacterial_contamination_gel.jpg; bacterial_contamination_reduction_gel.jpg; sispa_gel.jpg (Gel images)

Data are available under the terms of the Creative Commons Attribution 4.0 International license (CC-BY 4.0).
