# Supplementary material for: Enrichment approach for unbiased sequencing of respiratory syncytial virus directly from clinical samples
Source: Wellcome Open Res. 2021 May 7;6:99. [Version 1] doi: 10.12688/wellcomeopenres.16756.1 (PMC11109592; doi:10.12688/wellcomeopenres.16756.1)
Supplement: Peer review history

# Reviewer report: Reviewer response for version 1

**DOI:** 10.21956/wellcomeopenres.18478.r80061

**Published:** 2024-05-20

**Authors:** Tanya Golubchik

**Affiliations:** 1 Nuffield Department of Medicine, University of Oxford, Oxford, UK; 2 School of Medical Sciences, Faculty of Medicine and Health, University of Sydney, Sydney, Australia

The authors report on a series of optimisation experiments to identify a protocol capable of recovering complete or near-complete genomes of respiratory syncytial virus (RSV) from clinical respiratory samples. The experiments are well conducted and clearly described. Although the results are not positive, useful information is reported on the relative efficiency of sample purification steps. These include some limited centrifugation experiments, and DNAse treatment of whole or pre-centrifuged samples. The experimental design is complicated by an attempt to also test the suitability of the SISPA protocol for nanopore (ONT) sequencing, which the authors report to be biased to some RSV sequence motifs.

 The major findings were that (1) relatively brief centrifugation (5 min at 8000 rpm) resulted in co-precipitation of virus with host and bacterial cell debris, although sufficient virus remained in the supernatant to proceed; (2) precipitation of virus from the remaining supernatant (60min at 14k rpm) recovered RSV in the concentrate at Ct values comparable to starting samples, albeit with reduction in volume, and (3) DNAse digestion prior to RNA extraction drastically reduced RSV viral load, from Ct 24 (which would be sufficient for full genome recovery by most methods), to >Ct34, beyond the scope of most known protocols to recover whole viral genomes. The sequencing results, using SISPA random primers followed by ONT, did not yield appreciable RSV genome coverage with any protocol.

 Major comments:

- No positive controls (RSV or other) are shown for which complete genomes might have been obtained with the selected sequencing approach, so it is not clear to what extent SISPA+ONT would have been an unsuitable method even in the presence of high quality starting material. Did the authors have success with SISPA on any other samples?
- It is not clear from the report to what extent RNA was preserved when the DNAse treatment step was moved to “just prior to RNA extraction”. Figure 5 shows near-complete abolishment of RSV viral load in the DNAse-treated samples, but the text only states that motivated by this, the timing of the DNAse treatment step was changed. These results should be reported in Fig 5 as well, particularly as it is this version that is then sequenced.
- The aim of the experiments as stated was to design a procedure suitable for direct-from-sample metagenomics of viruses. RSV was presumably chosen because it is a known clinically important virus, which could be readily detected and quantified to assess method performance. However, the experimental design was targeted specifically at RSV, and no attempt is reported to track the fate of other viruses through the various steps - thus, the findings are RSV-specific. The introduction should make this clear.
- Given the experimental design is focused on RSV recovery, did the authors attempt any of the multiple previously published RSV-specific protocols for sequencing, in preference to SISPA? In the simplest case, published RSV-specific primers (eg Agoti et al J.Virol 2015) could have been used to produce amplicons, and this sequenced to demonstrate genome recovery, to serve as positive controls.
- Can the authors comment on the generalisability of any of the attempted protocols for viral metagenomics beyond RSV?
- A wealth of literature on centrifugation for virus recovery exists, and all protocols highlight the propensities of different viruses to be recovered in various fractions, depending the size of virions and their sedimentation rate (S value). These protocols often involve a combination of different gradients (surcrose, caesium chloride etc) to purify viruses from very large starting volumes of cell culture stock. Why was the specific centrifugation protocol in this study chosen? Would the authors see it as practical for respiratory samples in a clinical setting?

Minor comments: 

- The figure resolution is too low in the version reviewed, particularly Figure 2 and Figure 5.

Is the work clearly and accurately presented and does it cite the current literature?

Yes

If applicable, is the statistical analysis and its interpretation appropriate?

Yes

Are all the source data underlying the results available to ensure full reproducibility?

Yes

Is the study design appropriate and is the work technically sound?

Yes

Are the conclusions drawn adequately supported by the results?

Yes

Are sufficient details of methods and analysis provided to allow replication by others?

Partly

Reviewer Expertise:

Pathogen genomics, Bioinformatics, Computational microbiology, Viral and bacterial sequencing

I confirm that I have read this submission and believe that I have an appropriate level of expertise to confirm that it is of an acceptable scientific standard, however I have significant reservations, as outlined above.

# Reviewer report: Reviewer response for version 1

**DOI:** 10.21956/wellcomeopenres.18478.r46402

**Published:** 2024-05-11

**Authors:** Gerald Mboowa

**Affiliations:** 1 The African Center of Excellence in Bioinformatics and Data-Intensive Sciences, The Infectious Disease Institute, Makerere University, Kampala, Uganda

The article describes an enrichment approach for unbiased sequencing of respiratory syncytial virus directly from clinical samples. Suggestions within sentences are given in square brackets.

**Abstract:**

**Methods**

- Assessed two protocols using RSV positive samples. Clearly indicate whether these were fresh or preserved archived samples?Protocol 1 include physical pre-treatment enrichment centrifugal i.e the details such as speed, time, and temperature.
  Concentrates from Protocols 1 & 2 were each divided into two fractions; one was DNase treated while the other was not. How was normalization between DNase treated and untreated samples from the two protocols done? 

**Conclusions**

- 'We attribute the incomplete genome segments to amplification biases resulting from the use of short length random sequence (6 bases) in tagged Endoh primers. Increasing the length of the random nucleotides from six hexamers to nine or 12 in future studies may reduce the coverage biases' Did the authors mean improve capture and coverage of the template?

**Introduction:**

- 'The virus also causes high morbidity and mortality among immunocompromised individuals and the elderly (Englund et al., 1991' This reference is too outdated.

**Methods:**

- Study samples 'transported to KEMRI-Wellcome Trust Research Programme laboratories [within] four hours after collection where they were stored at -80°C.'

**Ethical considerations:**

- 'The study was ethically approved by the Kenya Medical Research Institute (KEMRI) Scientific and Ethics Review Unit (SERU #3103)' Include the date of this approval.
- Written informed consent had been collected from all the patient caregivers before using the samples for this study. I think it is important that authors indicate that they sought waiver of consent to use these samples from the IRB because assent and consent would be possible to be obtained from the study participants.

**Protocol 1: Centrifugal processing approach:**

**Optimization:**

- 'A set of 12 RSV positive samples were used at first to optimize the centrifugal pre-processing protocol. The protocol involved centrifugation of 400μl of sample at 8000 rpm for 5 minutes, which resulted in a pellet constituted mainly of the dense host and bacterial content. A volume of 350μL supernatant was collected and transferred to the 3kD Scientific Centrifugal Filter (Thermo Fischer)...' This should be 'Thermo Fisher Scientific', check this spelling throughout the article. Also include vendor’s the city and country, same for Beckman Coulter.
- 'The effectiveness of the pre-processing steps was assessed by performing RNA HS (high sensitivity) qubit, multiplex RT- PCR and IFAT.' Write IFAT in full.

**Bioinformatics analysis:**

- 'The reads generated from both protocols were taken through bioinformatics analysis using open-source tools other than for the Guppy base-calling software (version 3.1.5, ONT technologies). An alternative open-source base calling tool is Scrappie (https://github.com/nanoporetech/scrappie), a technology illustrator also developed by ONT community.' Did the authors call the bases using guppy or scrappie? Otherwise, this statement is not necessary if they called bases using Guppy as seen in the subsequent sentence.
- 'All the reads that passed QC (Phred score >7) test were then mapped to the corresponding 12 RSV references generated from Illumina'. Authors should include the reference or source where these 12 genomes can be located.

**Results:**

**Protocol 1: Centrifugal processing approach optimization**

- 3.1.1: Optimization. After comparing the RNA Qubit scores, cycle threshold (Ct) scores and IFAT images from the concentrate, filtrate and pellet (Waweru et al., 2021), we observed that nucleic acid content in the concentrate and filtrate was undetectable compared to the pellet (Figure 2A). The filtrate was RSV negative suggesting little or no virus loss during centrifugal filtration while the pellet had a lower Ct score than the concentrate suggesting more viral content [quantity] in the pellet relative to the concentrate (Figure 2B). Samples taken through direct RNA extraction as described in Protocol 2 but not treated with DNAse termed as typical RSV positive samples here, had comparable Ct scores to the concentrates (Figure 2B).

**Protocol 1: Centrifugal processing results:**

- We recovered 8.2 million reads from this protocol, 7.2 million of which passed quality check (QC) [abbreviate this once] with their median read quality being 11.11. Taxonomic classification of all the reads that passed QC from this protocol using Kraken2 indicated.

Is the work clearly and accurately presented and does it cite the current literature?

Yes

If applicable, is the statistical analysis and its interpretation appropriate?

Yes

Are all the source data underlying the results available to ensure full reproducibility?

Yes

Is the study design appropriate and is the work technically sound?

Yes

Are the conclusions drawn adequately supported by the results?

Yes

Are sufficient details of methods and analysis provided to allow replication by others?

Yes

Reviewer Expertise:

Bioinformatics

I confirm that I have read this submission and believe that I have an appropriate level of expertise to confirm that it is of an acceptable scientific standard.

# Reviewer report: Reviewer response for version 1

**DOI:** 10.21956/wellcomeopenres.18478.r47101

**Published:** 2022-01-31

**Authors:** Martin M. Nyaga, Milton Mogotsi

**Affiliations:** 1 Next Generation Sequencing Unit, Division of Virology, Faculty of Health Sciences, University of the Free State, Bloemfontein, South Africa; 2 Next Generation Sequencing Unit and Division of Virology, Faculty of Health Sciences, University of the Free State, Bloemfontein, South Africa

In the study “Enrichment approach for unbiased sequencing of respiratory syncytial virus directly from clinical samples” by Waweru *et al*., 2021, the authors attempted to develop a metagenomics protocol for enrichment of respiratory syncytial virus (RSV), which entailed centrifugal filtration, nuclease treatment and random amplification.

 This is a very impressive study that was thoroughly performed, and the authors compared their results extensively with the relevant international literature on the subject. Below are several comments and suggestions from the reviewer.

 The authors described that their enrichment procedure in “protocol 1” entailed centrifugation and filtration of the RSV positive samples. The resulting pellet following centrifugation and the filtrate and concentrate resulting from filtration were all subjected to RNA extraction.

 The authors report that after comparing the RNA Qubit scores, Ct values and immunofluorescence images of the antibody tests, the nucleic acid content of the concentrate and filtrate was undetectable compared to the pellet. This is expected since a larger fraction of the cells (host and bacterial cells), and therefore nucleic material, will be retained in the pellet.

 The authors should take into account that centrifugation conditions can also influence the reduction of viral particles and non-viral particles such as bacteria. Centrifugation is expected to pellet larger particles, while viruses remain in solution. Although procedures differ among studies, for a more thorough optimization, authors could also look into testing and comparing different centrifugation speeds and times to determine which conditions yield better results (improved viral recovery).

 After subjecting the supernatant to filtration using a 3kD centrifugal filter, the authors stated that the filtrate was RSV negative suggesting no virus loss during filtration. In my understanding, it seems the aim here was to retain the viruses in the “concentrate”, i.e., to prevent virus particles from passing through the filter.

 I think it would have been more efficient if the authors opted to collect virus particles in the “filtrate” rather than the “concentrate” being the main fraction of focus since viruses are generally smaller in size and therefore expected to easily pass through the filter. However, with that approach, it is advisable to filters of different particle sizes with indicated pore-sizes (0.22 µm, 0.45 µm, 0.8 µm), preferably 0.22 µm since the RSV virion ranges from 150-250nm in diameter. The majority of the larger eukaryotic and prokaryotic cells are not expected to pass through the filter, thereby enhancing viral recovery. Additionally, authors must also consider subjecting the sample to homogenisation prior to centrifugation. A non-homogenous suspension can result in clogging of the filter. This should, however, be done carefully as it may result in the destruction of virus particles (must be done at the appropriate speed, and without beads).

 After sequencing, the authors report that 90% of the 45 000 reads generated belonged to bacteria and host. As a result, the experiment was repeated but this time around the concentrate was subjected to DNase treatment to remove genomic DNA. The authors reported that after analysis 16s rRNA, the bacterial contamination was still very high. The reason could be that the bacterial genome is intact within the bacterial cell, the DNase treatment can only digest the free-floating nucleic material.

 As mentioned by the authors, it is crucial to include a DNase treatment to remove host and bacterial contaminants, however, due to the larger sizes of host and bacterial genomes compared to that of viruses, a bulk of host and bacterial reads will be recovered even after enrichment, dominating the sequence data.

 Overall, this was a very interesting study, the authors have done excellent work. The results and discussion parts are well described and were able to sufficiently support their findings with literature/previous studies. There are no major issues to be addressed by the authors.

Is the work clearly and accurately presented and does it cite the current literature?

Yes

If applicable, is the statistical analysis and its interpretation appropriate?

Not applicable

Are all the source data underlying the results available to ensure full reproducibility?

Yes

Is the study design appropriate and is the work technically sound?

Yes

Are the conclusions drawn adequately supported by the results?

Yes

Are sufficient details of methods and analysis provided to allow replication by others?

Yes

Reviewer Expertise:

Virology; Next Generation Sequencing; Molecular Biology

We confirm that we have read this submission and believe that we have an appropriate level of expertise to confirm that it is of an acceptable scientific standard.
